# Changes in Novel Anthropometric Indices of Abdominal Obesity during Weight Loss with Selected Obesity-Associated Single-Nucleotide Polymorphisms: A Small One-Year Pilot Study

**DOI:** 10.3390/ijerph191811837

**Published:** 2022-09-19

**Authors:** Katarzyna Iłowiecka, Paweł Glibowski, Justyna Libera, Wojciech Koch

**Affiliations:** 1 Department of Food and Nutrition, Medical University of Lublin, 4a Chodźki Str., 20-093 Lublin, Poland; 2 Department of Biotechnology, Microbiology and Human Nutrition, University of Life Science in Lublin, 8 Skromna Str., 20-704 Lublin, Poland; 3 Division of Engineering and Cereals Technology, Department of Plant Food Technology and Gastronomy, University of Life Sciences, 8 Skromna Str., 20-704 Lublin, Poland

**Keywords:** obesity, weight loss, anthropometric measurements, anthropometric indices, single-nucleotide polymorphisms

## Abstract

Whether BMI and the competing waist circumference (WC)-based anthropometric indices are associated with obesity-related single-nucleotide polymorphisms (SNPs) is as yet unknown. The current study aimed to evaluate the anthropometric indices (fat mass index, body shape index, visceral adiposity index, relative fat mass, body roundness index, and conicity index) during a weight loss intervention in 36 obese individuals. Blood biochemical parameters (total cholesterol, low-density lipoprotein, high-density lipoprotein, and triglycerides) and three SNPs (FTO rs9939609, TFAP2B rs987237, and PLIN1 rs894160) were assessed in 22 women and 14 men (35.58 ± 9.85 years, BMI 35.04 ± 3.80 kg/m^2^) who completed a 12-month balanced energy-restricted diet weight loss program. Body composition was assessed via bioelectrical impedance (SECA mBCA515). At the end of the weight loss intervention, all anthropometric indices were significantly reduced (*p* < 0.05). For the SNP FTO rs9939609, the higher risk allele (A) was characteristic of 88.9% of the study group, in which 10 participants (27.8%) were homozygous. We found a similar distribution of alleles in TFAP2B and PLIN1. Heterozygous genotypes in FTO rs9939609 and TFAP2B rs987237 were predisposed to significant reductions in WC-based novel anthropometric indices during weight loss. The influence of PLIN1 rs894160 polymorphisms on the changes in the analyzed indices during weight loss has not been documented in the present study.

## 1. Introduction

According to the World Health Organization (WHO), obesity management and the treatment of its complications are considered one of the most important public health challenges of the twenty-first century [1]. The reasons for the rapidly rising obesity prevalence are multifactorial. Among them, the most frequently mentioned are interactions between predisposing genetic and metabolic factors and the rapidly changing “obesogenic” environment [2]. Nowadays, obesity is examined as a multidimensional disease, in the therapy of which physical activity, dietary, behavioral, psychological, and pharmacological aspects play crucial roles [3,4,5,6]. 

The use of genetic tests in daily nutritional clinical practice regarding polygenic obesity is not yet clearly defined. However, tests based on modern genetic technologies for personalized dietary practice (direct-to-consumer tests, or DTC) are gaining popularity [7]. Dietary recommendations, in this case, are based on a genetic profile, taking into account SNPs marked for an individual patient. The clinical usefulness and benefits for patients using the DTC are limited and debatable, due to the high risk of incorrect interpretation of the test results [8]. Current research-based papers emphasize the need to conduct scientific investigations in the nutrigenetics field to develop an evidence-based approach, with strict regulations regarding the use of genetic tests to improve the personalized treatment of obesity [8,9].

In clinical practice, the most common methods of assessing nutritional status and general health conditions are simple anthropometric measurements, mainly based on the determination of body weight and height [10,11]. The current obesity classification based on body mass index (BMI), although useful for population studies, has significant limitations when applied to individuals [12]. Basically, this indicator does not take into account variables such as body composition, gender, age, ethnicity, or the presence of co-morbidities [13,14]. The incorrect categorization of obese individuals as healthy (and inversely) may lead to incorrect decisions during patients’ therapy [15]. For this reason, new obesity classifications are developing that consider additional, crucial aspects of obesity care, such as: clinical evaluation, diagnosis, obesity-related complications (ORC), therapy selection, treatment objectives, and options. One of the most important positions of recent years was proposed in 2016 by AACE/ACE (American Association of Clinical Endocrinologists/American College of Endocrinology) experts. The AACE/ACE obesity guidelines are as follows: obesity stage 0-BMI ≥ 30.0 kg/m^2^ and no ORC; obesity stage I-BMI ≥ 25.0 kg/m^2^ and one or more mild-to-moderate ORC; obesity stage II-BMI ≥ 25.0 kg/m^2^ and one or more severe ORC. Obesity-related complications include, e.g., metabolic syndrome, dyslipidemia, hypertension, obstructive sleep apnea, non-alcoholic fatty liver, or type 2 diabetes mellitus [16].

Regarding anthropometric parameters, simple and inexpensive tools are constantly sought that would allow clinicians to assess the content of body fat tissue and the risk of obesity-related metabolic diseases. The most notable of these tools are: the fat mass index (FMI), body shape index (ABSI), visceral adiposity index (VAI), relative fat mass (RFM), body roundness index (BRI), and conicity index (CI) [17,18,19,20,21]. In recent years, the association between these indices, with mortality [22] and many chronic diseases, e.g., metabolic syndrome [23,24,25], diabetes mellitus [26], hypertension [27,28], or cardiovascular risk [20], has been demonstrated. Moreover, novel anthropometric indices frequently showed a greater diagnostic ability of visceral or sarcopenic obesity and comorbidities, compared to BMI [26,29,30,31].

Changes in BMI and waist circumference (WC) are two of the main variables evaluated during the weight loss process [32,33]. Therefore, the relationship of these indicators with selected single-nucleotide polymorphisms (SNPs) related to obesity is widely described [2]. SNP-conditioned obesity (polygenic obesity) is the most common form of this disease, in terms of genetic factors [34]. Recent genome-wide association studies (GWAS) and whole-genome linkage analysis (genome-wide linkage studies, or GWLs) have revealed previously unknown loci and genes where the SNPs predispose to the development of obesity, to varying degrees. Scientific data regarding GWAS identified over 940 near-independent SNPs (discovered in more than 50 genes) related to BMI, of which FTO rs9939609 is the best known [35,36,37]. 

To the best of our knowledge, information regarding the association between novel anthropometric indices and genes that could play an important role in obesity development has not been previously described. As proved in the above paragraphs, there is a strong need to know these dependencies. The main objective of this research was to evaluate the values of BMI, FMI, ABSI, VAI, RFM, BRI, and CI during the weight loss process in obese individuals and estimate their associations with selected SNPs (FTO rs9939609, TFAP2B rs987237, and PLIN1 rs894160) related to excess body weight. 

## 2. Materials and Methods

The study was conducted according to the guidelines of the Declaration of Helsinki and approved by the Ethics Committee of the Medical University of Lublin (decision number: KE-0254/180/2019). The details of the test group, weight loss intervention, and outcome measurements have been described in our previous papers [38,39]. 

### 2.1. The Test Group

Briefly, the study was carried out in Lublin (the largest city in eastern Poland) on 36 Caucasian participants (22 women and 14 men) with an average age of 36.58 ± 9.85 years old. Potential participants were recruited through online and printed advertisements and were then assessed for the intervention by a dietitian (K.I.). The main inclusion criteria were: of adult age and obesity grade 0, according to the AACE/ACE classification [16], readiness for long-term dietary cooperation, and having given written permission to participate in the study. The exclusion criteria primarily included: pregnancy, contraindications to performing BIA (bioelectrical impedance analysis), obesity complications listed in the AACE/ACE classification, and other metabolic and chronic disorders.

### 2.2. Weight Loss Intervention

The 12-month dietary intervention was conducted at the Nutrition Center (University of Life Sciences in Lublin, Poland), and was designed in accordance with the position of the Polish Dietetic Association [40] and the Nutrition Standards for the Polish Population [41]. The nutritional guidelines for all participants met the qualitative and quantitative assumptions of a balanced diet, including an energy deficit in the range of 500–800 kcal/d. Furthermore, volunteers consumed four or five meals per day and were encouraged to eat a variety of vegetables, fruits, whole grain products, and home-cooked meals. An annual nutrition plan was developed individually for each participant, depending on their preferences and lifestyle.

The distribution of energy from proteins, fats, and carbohydrates was 15–25%, 25–35%, and 45–60%, respectively. The planned content of dietary fiber was at least 30 g/day. Furthermore, the intervention’s assumptions included limiting the daily energy supply from saturated fatty acids (<7%) and simple carbohydrates (<15%). The daily diet’s energy value was not to be lower than the resting metabolic rate (RMR) level calculated from the Mifflin-St. Jeor equation.

During the monthly follow-up visits, anthropometric measurements and BIA analysis were performed, and nutritional plans were discussed and modified if needed. Throughout the entire study, participants had unlimited access to support (e-mail or telephone) from a qualified dietitian.

### 2.3. Outcome Measurements

Anthropometric measurements and BIA analysis were performed at every follow-up visit during the research duration. Body height and WC were measured in a standing position, with an accuracy of 0.1 cm, via the wall-mounted stadiometer (SECA 216—seca GmbH & Co. KG., Hamburg, Germany) and stretch-resistant metric tape (SECA 201—seca GmbH & Co. KG., Hamburg, Germany), using standardized procedures [42,43]. Body weight was measured on a digital scale with a precision of 0.1 kg in lightweight clothes.

A SECA mBCA515 analyzer (seca GmbH & Co. KG., Hamburg, Germany) was used for body composition analysis using the BIA method (eight-point, multi-frequency impedance at 19 frequencies ranging from 1 to 1000 kHz), according to the manufacturer’s protocol. The main BIA exclusion criteria included: pregnancy, diagnosed epilepsy, implanted cardioverter or cardiac pacemaker, and the presence of metallic implants in the whole body (except dental). The analysis was performed in a fasting state (for at least 3 h before the measurement). Moreover, volunteers were recommended to refrain from intensive physical activity at least 12 h prior to testing and to empty their bladders 30 min prior to that. All measurements were performed using identical devices, in the same standardized conditions, by a single specialist (K.I.).

For each participant, three SNPs in three genes (FTO rs9939609, TFAP2B rs987237, PLIN1 rs894160) related to obesity were genotyped. Analyzed SNPs were identified using the polymerase chain reaction–restriction fragment-length polymorphism (PCR-RFLP) method. The genetic identification was carried out by an external laboratory (Vitagenum, Lublin, Poland). Genomic DNA was extracted from saliva using commercially available kits (Intergos, Legionów, Poland). Fasting blood samples: total cholesterol (Total-chol), low-density lipoprotein (LDL), high-density lipoprotein (HDL), and triglycerides (TG) were collected and analyzed by an external, diagnostic laboratory (Centrum Diagnostyki Laboratoryjnej, Lublin, Poland).

### 2.4. Anthropometric Indices

Based on the anthropometric measures, BMI, FMI, ABSI, VAI, RFM, BRI, and CI values were estimated.

(1) BMI = $\frac{\mathrm{weight}\mathrm{value}\left(\mathrm{kg}\right)}{\mathrm{height}{\left(m\right)}^{2}}$; unit: kg/m^2^ [44].
  - BMI ranges: underweight < 18.5 kg/m^2^; normal weight 18.5–24.9 kg/m^2^; overweight 25.0–29.9 kg/m^2^; obesity class I 30.0–34.9 kg/m^2^; obesity class II 35.0–39.9 kg/m^2^; obesity class III ≥ 40.0 kg/m^2^ [44].
(2) FMI = fat mass(kg)height(m)2; unit: kg/m^2^ [45].
  - FMI ranges for males: fat deficit < 3.0 kg/m^2^; normal 3.0–6.0 kg/m^2^; excess fat > 6.0–9.0 kg/m^2^; obesity class I > 9.0–12.0 kg/m^2^; obesity class II > 12.0–15.0 kg/m^2^; obesity class III > 15.0 kg/m^2^.
  - FMI ranges for females: fat deficit < 5.0 kg/m^2^; normal 5.0–9.0 kg/m^2^; excess fat > 9.0–13.0 kg/m^2^; obesity class I > 13.0–17.0 kg/m^2^; obesity class II > 17.0–21.0 kg/m^2^; obesity class III > 21.0 kg/m^2^.
(3) ABSI = $\frac{\mathrm{WC}\left(m\right)}{{\mathrm{BMI}}^{2/3}{\left(\mathrm{kg}/m\right)}^{2}\times {\mathrm{heigh}}^{1/2}\left(m\right)}$; no unit [46]
  - The proposed ABSI cut-off points in the diagnosis of metabolic disorders are 0.080 for males and 0.076 for females [47].
(4) VAI; no unit [48].
  Males: VAI = $\frac{\mathrm{WC}\left(\mathrm{cm}\right)}{39.68+(1.88\times \mathrm{BMI}\left(\mathrm{kg}/m^{2}\right)}\times \frac{\mathrm{TG}\left(\mathrm{mmol}/L\right)}{1.03}\times \frac{1.31}{\mathrm{HDL}\left(\mathrm{mmol}/L\right)}$
  Females: VAI = $\frac{\mathrm{WC}\left(\mathrm{cm}\right)}{36.58+(1.89\times \mathrm{BMI}\left(\mathrm{kg}/m^{2}\right)}\times \frac{\mathrm{TG}\left(\mathrm{mmol}/L\right)}{0.81}\times \frac{1.52}{\mathrm{HDL}\left(\mathrm{mmol}/L\right)}$
  The proposed VAI cut-off points identify a visceral adipose dysfunction (VAD) associated with cardiometabolic risk:
  - Adults < 30 years old: VAD absent ≤ 2.52; mild VAD 2.53–2.58; moderate VAD 2.59–2.73; severe VAD > 2.73.
  - Adults 30–41 years old: VAD absent ≤ 2.23; mild VAD 2.24–2.53; moderate VAD 2.54–3.12; severe VAD > 3.12.
  - Adults 42–51 years old: VAD absent ≤ 1.92; mild VAD 1.93–2.16; moderate VAD 2.17–2.77; severe VAD > 2.77 [49].
(5) RFM = 64 − (20 × $\frac{\mathrm{height}\left(m\right)}{\mathrm{WC}\left(m\right)})$ + 12 × gender (0 for males, 1 for females); unit: % [50].
  - The proposed RFM cut-off points to diagnose obesity and identify patients at higher risk of mortality are 30.0% for males and 40.0% for females [51].
(6) BRI = 364.2 – 365.5 $\times \sqrt{1–\;\left(\frac{\mathrm{WC}\left(m\right)/\left(2\pi \right){)}^{2}}{(0.5\times \mathrm{height}{\left(m\right)}^{2}}\right)}$; no unit [17].
  - The suggested BRI cut-off points to diagnose metabolic syndrome are 5.69 for males and 5.77 for females [52].
(7) CI = $\frac{\mathrm{WC}\left(m\right)}{0.109\sqrt{\frac{\mathrm{weight}\mathrm{value}\left(\mathrm{kg}\right)}{\mathrm{height}\left(m\right)}}}$; unit: m^3/2^/kg^1/2^ [53].
  - The suggested CI cut-off points to diagnose obesity and metabolic abnormalities are 1.200 for males and 1.180 for females [54].

### 2.5. Statistical Analysis

Statistical analysis was conducted for all variables. The distribution normality of the measurable variables was evaluated using the Shapiro–Wilk test (*p* < 0.05). A one–way analysis of variance (ANOVA) using Tukey’s post–hoc (*p* < 0.05) test was applied for the comparison of independent quantitative variables among more than two groups. The homogeneity of variance was assessed using Levene’s or Brown–Forsythe tests for the equality of variances. In the case of non-normal distributions, the nonparametric Kruskal–Wallis test (*p* < 0.05) was used. 

An ANOVA test with repeated measures (by Tukey’s post–hoc test, *p* < 0.05) was used to compare more than two dependent average values. Mauchly’s sphericity test was used to validate a repeated-measures analysis of variance. 

Changes in VAI values and blood biochemical parameters were compared using Student’s *t*-test or the Mann–Whitney U test (*p* < 0.05). A Pearson correlation between anthropometric indices and blood biochemical parameters was evaluated (*p* < 0.05; *p* < 0.001). The analyses were performed using STATISTICA 13.3 computer software (StatSoft, Inc., Tulsa, OK, USA).

## 3. Results

A total of 36 obese participants (22 women and 14 men) were included in the intervention. As shown in Table 1, the annual weight loss process has contributed to a significant improvement in most anthropometric parameters. The presented results indicate that the general trend was to recover or reverse at 12 months part of the beneficial effects achieved at 6 months on the studied parameters. The values of phase angle, HDL, and TG did not change statistically significantly after 12 months. During the weight loss phase, an increase in LDL and total-chol values were documented.

The initial assumptions of the study adopted only two control points: at the beginning (baseline) and at the end of the weight loss phase (12th month). During follow-up visits, results from BIA, WC measurements, and blood analyses were collected. However, our analysis of the results obtained after the end of the study prompted us to publish the BIA results after 6 months to show more comprehensive changes in body composition during the intervention. These are additional results. For this reason, the other outcomes from the sixth month were not presented. Importantly, the characteristics of the studied group and the other outcomes presented in Table 1 have been published in our previous work [39]. The percentage of subjects in which a reduction (>5%) and a regaining of initial body weight were observed at 6 and 12 months of intervention and the results are presented in Table S1 of the Supplementary Materials.

Based on the above parameters, the BMI, FMI, ABSI, VAI, RFM, BRI, and CI values were estimated. Changes in anthropometric indices during the weight loss phase in the study group are presented in Table 2.

Each of the analyzed indices had improved significantly at the end of the intervention. The results of the BMI, FMI, and RFM tests, obtained in the sixth month, were significantly more favorable compared to the results at the end of the research. The presented outcomes demonstrate that after reaching a plateau or a decline at 6 months, some indices recover part of the initial values. However, these results did not return to the baseline point.

Table 2 also presents an ABSI z-score indicator determining premature mortality risk levels. The results are classified into five categories: (1) very high: > 0.798; (2) high: 0.798–0.229; (3) average: 0.229–−0.272; (4) low: −0.272–−0.868; (5) very low: < −0.868. In the study group, the risk of premature death was reduced from the low to the very low range [46]. 

Pearson’s correlation between the anthropometric indices and selected blood parameters was analyzed (Table 3). The greatest number of significant associations was indicated for: WC, weight value, BMI, BRI, and VAT. The highest number of negative correlations was demonstrated for HDL, of which five (for weight, VAT, WC, BMI, and VAI) were statistically significant. Correlations between FM% and other indicators were not significant, except for FMI (r = 0.876; *p* < 0.001), and RFM (r = 0.830; *p* < 0.001). Likewise, only two significant associations, between TG, VAI (r = 0.815; *p* < 0.001) and WC (r = 0.335; *p* < 0.05), were noticed.

In the current small sample size study, ABSI was found to be independent of BMI (r = 0.168). The other WC-based central body indices in fact showed higher correlations: BRI r = 0.801, CI = r0.449, VAI r = 0.445, RFM r = 0.283. Therefore, ABSI provides the central body measure that is least dependent on BMI. 

For each participant, three SNPs in three genes (FTO rs9939609, TFAP2B rs987237, PLIN1 rs894160) related to obesity were genotyped. The frequency of alleles for the analyzed SNPs is summarized in Table 4. 

The TFAP2B and PLIN1 genes showed similar genotype frequencies. The smallest percentage of the group was characterized by an unfavorable SNP variant (11.1% and 5.6% in TFAP2B and PLIN1, respectively). Different results were noted regarding FTO, which is considered the main gene associated with obesity. The risk allele (A) was characteristic of 88.9% of the study group, in which 10 participants (27.8%) were discovered with the variant most at risk (AA). The opposite TT genotype was characterized by only 11.1% of patients. SNPs distribution in the study group has been published in our previous work [39].

Table 5, Table 6 and Table 7 summarize the results of changes in the anthropometric indices depending on analyzed SNPs. These analyses were conducted to evaluate whether individual SNPs can affect the anthropometric indices during the weight loss process. 

After the initial rapid improvement in BMI value (−1.95 kg/m^2^; −5.43%) in participants with the AA genotype, the results began to change adversely in the second part of the intervention and returned to the level before the weight loss process (−0.50 kg/m^2^; −1.39%). Similarly, FMI and VAI values did not differ significantly at the beginning and after 12 months of intervention. The FMI value in the AA genotype in the twelfth month was higher compared to the beginning of the study by 0.10 kg/m^2^ (+0.69%), despite the implementation of a negative energy balance. Other indicators (ABSI, RFM, BRI, CI, WC) were significantly reduced. 

The best results were obtained in the heterozygous AT allele variant, where all anthropometric indices were significantly reduced after dietary intervention.

Interestingly, participants with the TT genotype (the most favorable variant, considering the low risk of obesity development) did not significantly reduce any parameters in the twelfth month. However, the presented outcomes may have been caused due to the small sample number (*n* = 4). 

The size of the changes between the same control points (baseline, the sixth month, and the twelfth month, indicated by capital letters) in all genotypes was similar in most of the analyzed indices. Significant differences were noticed only in the FMI and RFM values in the twelfth month. 

In participants with the GG genotype, only the BMI value was significantly reduced (−1.85 kg/m^2^; −4.81%) in the twelfth month. Other analyzed anthropometric indices had returned to the baseline. Regarding the heterozygous AG genotype, all parameters have been improved significantly. In the case of patients with the AA genotype, the majority of the parameters had improved (ABSI, RFM, BRI, CI, and WC). BMI, FMI, and VAI had not been significantly reduced in the twelfth month of the intervention (*p* > 0.05). The size of the changes between the same control points (baseline, sixth month, and twelfth month; capital letters) in all genotypes was the same in all analyzed indices. 

Due to the small number of participants with the AA genotype (*n* = 2) in the gene PLIN1 rs894160, two groups were compared, with allele A (AA, AG) and without (GG). The presence of risk allele A did not affect the size of the changes in anthropometric indices. Similar results were obtained in both analyzed groups. Almost all analyzed parameters in the twelfth month were significantly lower than at the beginning of the study (except for FMI in the GG genotype). Similarly, the size of the changes in the analyzed parameters in the sixth and twelfth months (capital letters) did not differ significantly in both groups.

## 4. Discussion

Previous studies evaluated the association of novel anthropometric indices with mortality and many obesity-related chronic diseases [20,22,23,24,25,26,27,28]. The present research is among the first intervention study estimating the relationships between novel anthropometric parameters and selected obesity-related genes. Due to the lack of data regarding the above issue (except for BMI associations), it is not possible to compare all the results of the present research with the other authors’ findings. Therefore, the discussion concerns mainly the correlation between the novel anthropometric indices and analyzed health indicators. Moreover, the genotype frequencies of FTO rs9939609, TFAP2B rs987237, and PLIN1 rs894160 were raised in this section.

The present research indicated that the frequency of the FTO rs9939609 was 27.8%, 61.1%, and 11.1% for the AA, AT, and TT genotypes, respectively, which was in conformity with the study by Abolnezhadian et al. [55]. The research performed by Prakash et al. [56] in an obese population showed slightly different results: 23.9%, 44.7%, and 31.4% for the AA, AT, and TT genotypes, respectively. Likewise, Mehrdad et al. [57] proved that in a group of overweight healthy adults, about half of the participants were AT genotype, 19% of them were AA variant, and approximately 31% of them were characterized by the TT homozygote. In addition, a study in the United Kingdom, conducted on an overweight population-based sample, documented similar results [58]. According to Stocks et al. [59], the smallest percentage (2.6%) of obese patients is characterized by the GG genotype in the TFAP2B (transcription factor AP-2 Beta) gene, which was confirmed by our results (11.1%). The obtained results reported that 44.4% of participants had an AG or AA genotype, while Stock et al. [59] indicated 23.50% and 53.63%, respectively. In line with our outcomes regarding the genotype frequencies of PLIN1 rs894160 were the studies carried out by Ruiz et al. [60], Qi et al. [61], and Meirhaeghe et al. [62]. In all the above studies, the AA genotype was characteristic of a slight percentage of respondents (5.6–12.8%). However, both the AG and GG variants were documented in over 40% of obese participants.

At the end of the weight loss intervention, all the anthropometric indices were significantly reduced (*p* < 0.05). Regarding biochemical parameters, an increase in LDL and total-chol values was documented, which may be a temporary state resulting from a change in the lipoprotein metabolism. Lipidogram values should decrease once the body weight is stabilized [63,64]. Numerous previous studies have demonstrated the role of dietary modifications in the regulation of BMI value [65]. Nevertheless, the problem is in discovering the genes and SNPs related to excessive body weight and defining by which mechanisms they exert their effect. The FTO (the fat mass and obesity-associated gene) has the greatest influence on BMI values of all known genes [66]. The association between FTO rs9930506 polymorphism and changes in BMI after lifestyle interventions has been studied frequently. Reinehr et al. [67] demonstrated that the obesity risk allele (A) was not associated with BMI decrease during a lifestyle intervention (*p* = 0.777), but the studied SNPs were associated with BMI regain after a one-year follow-up period. These results were in concordance with the results reported by Matsuo et al. [68] and Delahanty et al. [69], which showed no significant differences in the BMI value during the weight loss programs, regardless of rs9930506 SNP. Similar conclusions were also presented from studies conducted on children and adolescents [70,71]. Interestingly, some investigations have demonstrated contradictory outcomes, suggesting that individuals carrying the homozygous AA genotype had a greater BMI decrease than TT genotypes after nutrition interventions [72]. Although it seems that all patients, despite their genotype, can decrease their BMI significantly in response to a short-term lifestyle intervention; some of the results showed that rs9930506 AA-genotype individuals may have more difficulty reducing body fat than subjects with the other variants [73]. Our study indicated that the most favorable results were obtained in the heterozygous AT genotype, where a significant decrease in BMI was shown in the twelfth month of intervention, in contrast to the AA and TT variants.

In terms of the relationship between BMI and the PLIN1 rs894160 polymorphism, Ruiz et al. [60] found that SNPs in the analyzed genes were not associated with BMI changes during a 12-week energy-restricted diet intervention. The subjects (obese women) with genotypes GG, GA, and AA reduced their BMI value by −8.8 kg/m^2^, −8.9 kg/m^2^, and −7.6 kg/m^2^ respectively, which were not statistically significant differences (*p* = 0.488). The obtained outcomes are in accordance with the results of Soenen et al. [74], who indicated that BMI values were significantly lower for obese men with the A allele compared to those with the GG genotype (30.5 ± 1.4 kg/m^2^ vs. 33.5 ± 4.1 kg/m^2^, *p* < 0.01; in our study, the results were 33.68 kg/m^2^ ± 2.91 vs. 36.19 ± 4.10 kg/m^2^, *p* < 0.05). Moreover, the lower values of BMI that were associated with the AA and AG genotypes at baseline were still apparent during the two weight-loss control points. 

Only one clinical trial assessed the relationship between the ABSI value and the FTO gene. Ahmad et al. [75] indicated that FTO rs9939609 polymorphism was unrelated to the change in anthropometric status following epigallocatechin-gallate (EGCG) supplementation intervention. Contrastingly, our study documented that the AA and AT allele carriers significantly reduced ABSI values after 12 months of intervention, compared to individuals characterized by the homozygote TT. It should be remembered that the small sample size of the TT genotype (*n* = 4) raises uncertainty regarding the effects in this group. In their study, Abolnezhadian et al. [55] also examined the ABSI value and FTO rs9939609 SNPs, but the relationship between them was not analyzed. The observed lack of correlation between BMI and ABSI has been documented in previous papers [29,76].

Thomas et al. [17] created the BRI to estimate body fat mass, visceral adiposity percentage, and an individual’s physical health. In an overweight Chinese adult population, BRI was found to be correlated with HDL and TG after adjusting for gender and age [77]. These results have not been confirmed in our study (r = 0.281 and r = −0.270 respectively), or in the research conducted by Gomez-Marcos et al. [52] (r = −0.110 and r = 0.107, respectively). BRI was usually applied as an adipose indicator for diagnosing hyperuricemia [78], arterial stiffness [77], cardiometabolic risk [79], diabetes [80], hypertension [27], or metabolic syndrome risk [24]. 

The results of the present paper revealed a positive significant correlation between BMI and CI (r = 0.449). In line with our results are those reported by Shidfar et al. [81] (r = 0.310). A slightly weaker association has been demonstrated by Nkwana et al. [20] (r = 0.150 for men and r = 0.016 for women). The correlation between CI and lipidogram markers that was shown in the above-cited study was in accordance with our outcomes (r = 0.286 for TG and r= −0.248 for HDL). In general, CI is a straightforward method for evaluating abdominal obesity and also its relationship to cardiovascular risk factors [82]. 

The correlation between RFM and FM% (r = 0.830; *p* < 0.001) described in the present research was close to the values obtained by Corrêa et al. [83] (0.880; *p* < 0.05). However, the association between RFM and BMI was highly different: r = 0.283; *p* > 0.05 in our study, and r = 0.890; *p* < 0.05 in the results reported by Corrêa et al. [83]. Additionally, RFM had a highly significant positive correlation with FM% (r = 0.751, *p* < 0.001), and changes in RFM during the lifestyle intervention were significantly correlated with changes in FM% [84]. It has been shown that RFM can be a better predictor than BMI of dyslipidemia and metabolic syndrome [85], or of liver disease and mortality [86].

Anthropometric (BMI and WC) and functional (TG and HDL) parameters were used to calculate the VAI. This measure, which may be viewed as a straightforward surrogate marker of visceral adipose dysfunction, in previous studies demonstrated a significant correlation with VAT. Additionally, it indicated a strong independent association with cardiovascular events [48] and demonstrated a superior predictive ability for incident diabetic events, compared to its individual components (WC, BMI, TG, and HDL) [87]. The results obtained by Adanas and Özgen [88] proved that the correlations between VAI and BMI, TG, and HDL were r = 0.503, r = 0.909, r = −0.730, respectively, which is comparable to our results (r = 0.445, r = 0.815, r = −0.581, respectively). The association between VAI and BMI at around r = 0.500 also was confirmed in other studies [89], while different values were obtained by Zar and Ali [90] (r = −0.016; *p* = 0.788) and by Wanderley Rocha et al. [91] (r = 0.160; *p* > 0.05).

An FMI is the measure of relative fat content. Its independence from fat-free mass gives it a diagnostic edge over FM%. The obtained results indicate that FMI has the greatest correlations with FM%, BMI, and RFM (r = 0.876, r = 0.738, r = 0.740; *p* < 0.001, respectively), which finding is confirmed by previous studies [29,92].

To summarize the information presented above, the strongest Pearson’s rank correlations were obtained between BMI and BRI (r = 0.801; *p* < 0.001), FMI (r = 0.738; *p* < 0.001), CI (r = 0.449; *p* < 0.05), and VAI (r = 0.445; *p* < 0.05). In terms of WC and the novel anthropometric indices, highly significant associations were observed for: CI (r = 0.897; *p* < 0.001), BRI (r = 0.895; *p* < 0.001), ABSI (r = 0.786; *p* < 0.001), and the ABSI z-score (r = 0.697; *p* < 0.001). Therefore, it can eventually be suggested that BRI and CI may serve as comprehensive clinical indicators of obesity, as was confirmed in previous studies [80,93].

To the best of the authors’ knowledge, the present research is among the first clinical trials to evaluate changes in novel anthropometric indices during weight loss intervention and their associations with selected SNPs related to obesity. The other strengths of our study are its longitudinal design and the multiplicity of analyzed indices. The major limitation of the research is the small number of participants, whereas recent studies [94] and databases [95] have included thousands of subjects. Therefore, a full analysis of the results (including gender division) was not possible. Another weak point of the study was the incomplete data regarding biochemical blood analyses. During the study, participants were preparing meals themselves (without the researchers’ strict control), which could also have affected the obtained results. Additionally, due to the exclusion of participants with metabolic and autoimmune disorders, it can be difficult to generalize our findings to the average patient trying to seek medical help with weight loss.

## 5. Conclusions

The obtained results demonstrate that heterozygous genotypes in FTO rs9939609 and TFAP2B rs987237 predispose the subject to a significant improvement in novel anthropometric indices during weight loss. The influence of PLIN1rs894160 polymorphisms on the size of the changes in analyzed indices has not been documented. In a study group, the frequencies of carrying the riskiest genotype in relation to obesity development were the highest in the case of the FTO gene. Prospective studies with more patients, and with a greater number of analyzed genes, are needed to draw more accurate conclusions in the future. 

## Figures and Tables

**Table 1:** Characteristics of the studied group during the weight loss phase.

Parameter	Baseline	After 6 Months	After 12 Months
	*n* = 36 (*n* = 22 Women, *n* = 14 Men)		
Age (years)	36.58 ^B^ ± 9.85	37.14 ^AB^ ± 9.80	37.58 ^A^ ± 9.85
Height (m)	1.72 ^A^ ± 0.10	1.72 ^A^ ± 0.10	1.72 ^A^ ± 0.10
Body weight value (kg)	104.02 ^A^ ± 17.92	96.63 ^C^ ± 18.25	99.27 ^B^ ± 18.86
Fat mass (%)	41.11 ^A^ ± 5.96	38.23 ^C^ ± 6.64	39.40 ^B^ ± 10.46
VAT (L)	3.69 ^A^ ± 2.36	2.65 ^B^ ± 1.99	2.82 ^B^ ± 1.99
WC (cm)	107.72 ^A^ ± 13.56	99.42 ^C^ ± 13.50	101.17 ^B^ ± 13.34
Phase angle (°)	5.57 ^A^ ± 0.65	5.54 ^A^ ± 0.68	5.55 ^A^ ± 0.65
Total-chol (mmol/L)	4.92 ^B^ ± 0.93	nd	5.29 ^A^ ± 1.00
HDL (mmol/L)	1.37 ^A^ ± 0.38	nd	1.41 ^A^ ± 0.36
LDL (mmol/L)	2.98 ^B^ ± 0.80	nd	3.30 ^A^ ± 0.83
TG (mmol/L)	1.29 ^A^ ± 0.57	nd	1.27 ^A^ ± 0.45

Values with different letters in the same row are significantly different at *p* < 0.05 (ANOVA with repeated measures—Tukey test or Student’s *t*-test in biochemical parameters). Abbreviations: nd—no data, VAT—visceral adipose tissue, WC—waist circumference, Total-chol—total cholesterol, LDL—low-density lipoprotein, HDL—high-density lipoprotein, TG—triglycerides.

**Table 2:** Changes in anthropometric indices during the weight loss phase.

Anthropometric Indices	Baseline	After a 6 Months	After a 12 Months
BMI (kg/m^2^)	35.04 ^A^ ± 3.80	32.43 ^C^ ± 3.96	33.40 ^B^ ± 4.12
FMI (kg/m^2^)	14.46 ^A^ ± 3.03	12.47 ^C^ ± 3.13	13.35 ^B^ ± 3.37
ABSI	0.077 ^A^ ± 0.006	0.075 ^B^ ± 0.006	0.074 ^B^ ± 0.005
ABSI z-score	−0.459 ^A^ ± 1.156	−0.983 ^B^ ± 1.119	−1.108 ^B^ ± 0.929
VAI	1.74 ^A^ ± 0.97	nd	1.24 ^B^ ± 0.40
RFM (%)	39.38 ^A^ ± 5.73	36.62 ^C^ ± 5.88	37.28 ^B^ ± 5.86
BRI	6.13 ^A^ ± 1.57	5.02 ^B^ ± 1.41	5.25 ^B^ ± 1.39
CI (m^3/2^/kg^1/2^)	1.273 ^A^ ± 0.100	1.221 ^B^ ± 0.099	1.223 ^B^ ± 0.089

Values with different letters in the same row are significantly different at *p* < 0.05 (ANOVA with repeated measures—Tukey test or Student’s *t*-test). Abbreviations: nd—no data, BMI—body mass index, FMI—fat mass index, ABSI—A body shape index, VAI—visceral adiposity index, RFM—relative fat mass, BRI—body roundness index, CI—conicity index.

**Table 3:** Pearson’s rank correlation for body mass composition measurements and blood parameters at the baseline.

Parameter	Body Weight	FM%	VAT	WC	BMI	FMI	ABSI	ABSI z-s	VAI	RFM	BRI	CI	TG	HDL
**Body Weight**	−													
**FM%**	−0.141	−												
**VAT**	0.823 **	−0.320	−											
**WC**	0.837 **	−0.084	0.934 **	−										
**BMI**	0.768 **	0.328	0.598 **	0.754 **	−									
**FMI**	0.291	0.876 **	0.074	0.330 *	0.738 **	−								
**ABSI**	0.399 *	−0.281	0.774 **	0.786 **	0.240	−0.064	−							
**ABSI z-s**	0.286	−0.148	0.674 **	0.697 **	0.165	−0.002	0.949 **	−						
**VAI**	0.325	0.108	0.240	0.373 *	0.445 *	0.329	0.200	0.210	−					
**RFM**	−0.287	0.830 **	−0.367 *	−0.078	0.283	0.740 **	−0.144	−0.064	0.205	−				
**BRI**	0.589 **	0.194	0.749 **	0.895 **	0.801 **	0.550 **	0.715 **	0.660 **	0.392 *	0.298	−			
**CI**	0.541 **	−0.178	0.849 **	0.897 **	0.449 *	0.113	0.974 **	0.914 **	0.276	−0.072	0.843 **	−		
**TG**	0.301	−0.148	0.315	0.335 *	0.281	0.047	0.243	0.231	0.815 **	−0.042	0.281	0.286	−	
**HDL**	−0.464 *	0.190	−0.393 *	−0.392 *	−0.376 *	−0.069	−0.197	−0.148	−0.581 *	0.243	−0.270	−0.248	−0.243	−

* p < 0.05; ** *p* < 0.001; _, strong Pearson’s rank correlation > 0.7. Abbreviations: FM%—% fat mass, VAT—visceral adipose tissue, WC—waist circumference, BMI—body mass index, FMI—fat mass index, ABSI—A body shape index, VAI—visceral adiposity index, RFM—relative fat mass, BRI—body roundness index, CI—conicity index, TG—triglycerides, HDL—high-density lipoprotein.

**Table 4:** SNP distribution in the study group.

SNP Variant	Baseline (*n* = 36)
rs9939609 (FTO)	
AA *	10 (27.8%)
AT	22 (61.1%)
TT	4 (11.1%)
rs987237 (TFAP2B)	
GG *	4 (11.1%)
AG	16 (44.4%)
AA	16 (44.4%)
rs894160 (PLIN1)	
AA *	2 (5.6%)
AG	15 (41.7%)
GG	19 (52.7%)

* The most at-risk genotype.

**Table 5:** Differences in changes in the anthropometric indices, depending on FTO rs9939609.

Anthropometric Indices	rs9939609 (FTO)								
	AA (*n* = 10)			AT (*n* = 22)			TT (*n* = 4)		
	Baseline	6 Month	12 Month	Baseline	6 Month	12 Month	Baseline	6 Month	12 Month
BMI (kg/m^2^)	35.89 ^Aa^ ± 4.82	−1.95 ^Ab^ (−5.43%)	−0.50 ^Aab^ (−1.39%)	34.82 ^Aa^ ± 3.51	−3.01 ^Ac^ (−8.64%)	−2.24 ^Ab^ (−6.43%)	33.79 ^Aa^ ± 1.89	−2.26 ^Ab^ (−6.69%)	−0.90 ^Aab^ (−2.66%)
FMI (kg/m^2^)	14.42 ^Aa^ ± 3.55	−1.44 ^Ab^ (−9.99%)	0.10 ^Aa^ (+0.69%)	14.63 ^Aa^ ± 3.09	−2.25 ^Ab^ (−15.38%)	−1.69 ^Bb^ (−11.55%)	13.63 ^Aa^ ± 1.26	−1.99 ^Ab^ (−14.60%)	−0.99 ^ABab^ (−7.26%)
ABSI	0.079 ^Aa^ ± 0.004	−0.002 ^Aab^ (−2.53%)	−0.002 ^Ab^ (−2.53%)	0.076 ^Aa^ ± 0.006	−0.002 ^Ab^ (−2.63%)	−0.003 ^Ab^ (−3.94%)	0.076 ^Aa^ ± 0.008	−0.002 ^Aa^ (−2.63%)	−0.001 ^Aa^ (−1.32%)
VAI	1.67 ^Aa^ ± 0.97	nd	−0.32 ^Aa^ (−19.16%)	1.82 ^Aa^ ± 1.04	nd	−0.66 ^Ab^ (−36.26%)	1.49 ^Aa^ ± 0.64	nd	−0.07 ^Aa^ (4.70%)
RFM (%)	38.98 ^Aa^ ± 5.71	−1.99 ^Ab^ (−5.11%)	−1.22 ^Ab^ (−3.13%)	40.14 ^Aa^ ± 5.73	−3.18 ^Ab^ (−7.92%)	−2.64 ^Bb^ (−6.58%)	36.24 ^Aa^ ± 6.15	−2.36 ^Ab^ (−6.51%)	−1.38 ^ABab^ (−3.81%)
BRI	6.61 ^Aa^ ± 1.29	−0.86 ^Ab^ (−13.01%)	−0.57 ^Ab^ (−8.62%)	6.03 ^Aa^ ± 1.71	−1.26 ^Ab^ (−20.89%)	−1.09 ^Ab^ (−18.07%)	5.43 ^Aa^ ± 1.34	−0.90 ^Ab^ (−16.57%)	−0.49 ^Aab^ (−9.02%)
CI (m^3/2^/kg^1/2^)	1.309 ^Aa^ ± 0.062	−0.040 ^Ab^ (−3.05%)	−0.039 ^Ab^ (−2.98%)	1.259 ^Aa^ ± 0.108	−0.059 ^Ab^ (−4.69%)	−0.059 ^Ab^ (−4.69%)	1.261^Aa^ ± 0.128	−0.045 ^Aa^ (−3.57%)	−0.030 ^Aa^ (−2.38%)
WC (cm)	112.60 ^Aa^ ± 13.03	−6.30 ^Ab^ (−5.59%)	−4.10 ^Ab^ (−3.64%)	105.64 ^Aa^ ± 13.90	−9.41 ^Ab^ (−8.91%)	−8.18 ^Ab^ (−7.74%)	107.00 ^Aa^ ± 13.09	−7.25 ^Ab^ (−6.78%)	−3.75 ^Aab^ (−3.50%)

Values with different letters in the same row are significantly different at *p* < 0.05; ^abc^—differences between: baseline, 6th, and 12th month in a single genotype (ANOVA with repeated measures—Tukey test); ^AB^—differences between the same control points (baseline, 6th month, 12th month) in all genotypes (one–way ANOVA—Tukey test). Abbreviations: nd—no data, BMI—body mass index, FMI—fat mass index, ABSI—A body shape index, VAI—visceral adiposity index, RFM—relative fat mass, BRI—body roundness index, CI—conicity index, WC—waist circumference.

**Table 6:** Differences in changes in the anthropometric indices, depending on TFAP2B rs987237.

Anthropometric Indices	rs987237 (TFAP2B)								
	GG (*n* = 4)			AG (*n* = 16)			AA (*n* = 16)		
	Baseline	6 Month	12 Month	Baseline	6 Month	12 Month	Baseline	6 Month	12 Month
BMI (kg/m^2^)	38.44 ^Aa^ ± 4.68	−2.60 ^Ab^ (−6.76%)	−1.85 ^Ab^ (−4.81%)	33.83 ^Aa^ ± 3.27	−2.50 ^Ab^ (−7.39%)	−1.94 ^Ab^ (−5.73%)	35.31 ^Aa^ ± 3.62	−2.78 ^Ab^ (−7.87%)	−1.21 ^Aa^ (−3.43%)
FMI (kg/m^2^)	16.03 ^Aa^ ± 4.18	−1.78 ^Ab^ (−11.10%)	−0.65 ^Aab^ (−4.05%)	13.77 ^Aa^ ± 2.60	−1.83 ^Ab^ (−13.29%)	−1.49 ^Ab^ (−10.82%)	14.76 ^Aa^ ± 3.15	−2.21 ^Ab^ (−14.97%)	−0.85 ^Aa^ (−5.76%)
ABSI	0.076 ^Aa^ ± 0.006	−0.001 ^Aa^ (−1.32%)	−0.001 ^Aa^ (−1.32%)	0.075 ^Aa^ ± 0.007	−0.002 ^Ab^ (−2.67%)	−0.003 ^Ab^ (−4.00%)	0.078 ^Aa^ ± 0.004	−0.002 ^Ab^ (−2.56%)	−0.002 ^Ab^ (−2.56%)
VAI	2.01 ^Aa^ ± 1.19	nd	−0.68 ^Aa^ (−33.83%)	1.80 ^Aa^ ± 0.92	nd	−0.60 ^Ab^ (−33.33%)	1.61 ^Aa^ ± 1.02	nd	−0.36 ^Aa^ (−22.36%)
RFM (%)	38.82 ^Aa^ ± 6.24	−2.09 ^Ab^ (−5.38%)	−1.50 ^Aab^ (−3.86%)	38.78 ^Aa^ ± 5.86	−2.91 ^Ab^ (−7.50%)	−2.60 ^Ab^ (−6.70%)	40.12 ^Aa^ ± 5.78	−2.78 ^Ac^ (−6.93%)	−1.76 ^Ab^ (−4.39%)
BRI	6.60 ^Aa^ ± 1.56	−0.89 ^Ab^ (−13.48%)	−0.62 ^Aab^ (−9.39%)	5.62 ^Aa^ ± 1.59	−1.09 ^Ab^ (−19.39%)	−0.98 ^Ab^ (−17.44%)	6.51 ^Aa^ ± 1.50	−1.19 ^Ab^ (−18.28%)	−0.85 ^Ab^ (−13.06%)
CI (m^3/2^/kg^1/2^)	1.277 ^Aa^ ± 0.10	−0.034 ^Aa^ (−2.34%)	−0.023 ^Aa^ (−1.56%)	1.244 ^Aa^ ± 0.12	−0.056 ^Ab^ (−4.52%)	−0.060 ^Ab^ (−4.76%)	1.301 ^Aa^ ± 0.074	−0.053 ^Ab^ (−4.07%)	−0.048 ^Ab^ (−3.9%)
WC (cm)	115.00 ^Aa^ ± 13.09	−6.75 ^Ab^ (−5.87%)	−4.75 ^Aab^ (−4.13%)	103.00 ^Aa^ ± 14.30	−8.25 ^Ab^ (−8.01%)	−7.63 ^Ab^ (−7.41%)	105.00 ^Aa^ ± 17.06	−8.75 ^Ab^ (−8.33%)	−5.94 ^Ab^ (−5.66%)

Values with different letters in the same row are significantly different at *p* < 0.05; ^abc^—differences between: baseline, 6th, and 12th month in a single genotype (ANOVA with repeated measures—Tukey test); ^A^—differences between the same control points (baseline, 6th month, 12th month) in all genotypes (one–way ANOVA—Tukey test). Abbreviations: nd—no data, BMI—body mass index, FMI—fat mass index, ABSI—A body shape index, VAI—visceral adiposity index, RFM—relative fat mass, BRI—body roundness index, CI—conicity index, WC—waist circumference.

**Table 7:** Differences in changes in the anthropometric indices, depending on PLIN1 rs894160.

Anthropometric Indices	rs894160 (PLIN1)					
	AA + AG (*n* = 2 + 15)			GG (*n* = 19)		
	Baseline	6 Month	12 Month	Baseline	6 Month	12 Month
BMI (kg/m^2^)	33.68 ^Ba^ ± 2.91	−2.83 ^Ac^ (−8.40%)	−2.02 ^Ab^ (−6.00%)	36.19 ^Aa^ ± 4.10	−2.46 ^Ac^ (−6.80%)	−1.24 ^Ab^ (−3.43%)
FMI (kg/m^2^)	13.87 ^Aa^ ± 2.53	−2.03 ^Ab^ (−14.66%)	−1.41 ^Ab^ (−10.17%)	14.99 ^Aa^ ± 340	−1.96 ^Ab^ (−13.08%)	−0.85 ^Aa^ (−5.67%)
ABSI	0.076 ^Aa^ ± 0.007	−0.002 ^Ab^ (−2.63%)	−0.002 ^Ab^ (−2.63%)	0.077 ^Aa^ ± 0.005	−0.002 ^Ab^ (−2.60%)	−0.003 ^Ab^ (−3.90%)
VAI	1.69 ^Aa^ ± 0.95	nd	−0.48 ^Ab^ (−24.49%)	1.78 ^Aa^ ± 1.01	nd	−0.52 ^Ab^ (−29.21%)
RFM (%)	39.24 ^Aa^ ± 5.33	−2.99 ^A^^b^ (−7.62%)	−2.36 ^A^^b^ (−6.01%)	39.51 ^Aa^ ± 6.21	−2.55 ^Ab^ (−6.45%)	−1.88 ^Ab^ (−4.76%)
BRI	5.66 ^Aa^ ± 1.41	−1.08 ^Ab^ (−19.08%)	−0.88 ^Ab^ (−15.55%)	6.54 ^Aa^ ± 1.62	−1.14 ^Ab^ (−17.43%)	−0.88 ^Ab^ (−13.46%)
CI (m^3/2^/kg^1/2^)	1.254 ^Aa^ ± 0.111	−0.049 ^Ab^ (−3.91%)	−0.049 ^Ab^ (−3.91%)	1.291 ^Aa^ ± 0.088	−0.054 ^Ab^ (−4.18%)	−0.051 ^Ab^ (−3.95%)
WC (cm)	103.53 ^Aa^ ± 13.25	−8.29 ^Ab^ (−8.01%)	−6.94 ^Ab^ (−6.70%)	111.47 ^Aa^ ± 13.04	−8.32 ^Ab^ (−7.46%)	−6.21 ^Ab^ (−5.57%)

Values with different letters in the same row are significantly different at *p* < 0.05; ^abc^—differences between: baseline, 6th, and 12th month in a single genotype (ANOVA with repeated measures—Tukey test); ^AB^—differences between the same control points (baseline, 6th month, 12th month) in all genotypes (Student’s *t*-test or the Mann–Whitney U test). Abbreviations: nd—no data, BMI—body mass index, FMI—fat mass index, ABSI—A body shape index, VAI—visceral adiposity index, RFM—relative fat mass, BRI—body roundness index, CI—conicity index, WC—waist circumference.

## Data Availability

The data presented in this study are available on request from the first author (K.I.). The data are not publicly available due to restrictions concerning privacy.

## Supplementary Materials

The following supporting information can be downloaded at: https://www.mdpi.com/article/10.3390/ijerph191811837/s1: Table S1: The percentage of subjects in which the reduction (>5%) and regaining of initial body weight were observed.
