# Supplementary material for: Changes in Novel Anthropometric Indices of Abdominal Obesity during Weight Loss with Selected Obesity-Associated Single-Nucleotide Polymorphisms: A Small One-Year Pilot Study
Source: Int J Environ Res Public Health. 2022 Sep 19;19(18):11837. doi: 10.3390/ijerph191811837 (PMC9517315; doi:10.3390/ijerph191811837)
Supplement: Supplementary file 1 [file ijerph-19-11837-s001.zip › ijerph-1916903-supplementary.pdf]

# Changes in Novel Anthropometric Indices of Abdominal Obesity during Weight Loss with Selected Obesity-Associated Single-Nucleotide Polymorphisms: A Small One-Year Pilot Study

**Katarzyna Iłowiecka [1,*], Paweł Glibowski [2], Justyna Libera [3] and Wojciech Koch [1,*]**

[1] Department of Food and Nutrition, Medical University of Lublin, 4a Chodźki Str., 20-093 Lublin, Poland
[2] Department of Biotechnology, Microbiology and Human Nutrition, University of Life Science in Lublin, 8 Skromna Str., 20-704 Lublin, Poland
[3] Division of Engineering and Cereals Technology, Department of Plant Food Technology and Gastronomy, University of Life Sciences, 8 Skromna Str., 20-704 Lublin, Poland
* Correspondence: katarzyna.ilowiecka@umlub.pl (K.I.); kochw@interia.pl (W.K.); Tel.: +48-81-448-7142 (W.K.)

**Table S1.** The percentage of subjects in which reducing (>5%) and regain of initial body weight was observed.

| SNP Variant | Body Weight Improve in 6th Months | Body Weight Improve in 12th Months | Body Weight Regain in 12th Months |
|---|---|---|---|
| | | rs9939609 (FTO) | |
| AA (*n* = 10) | 5 (50.0%) | 1 (10.0%) | 6 (60.0%) |
| AT (*n* = 22) | 17 (77.3%) | 6 (27.3%) | 6 (27.3%) |
| TT (*n* = 4) | 4 (100.0%) | 2 (50.0%) | 1 (25.0%) |
| | | rs987237 (TFAP2B) | |
| GG (*n* = 4) | 3 (75.0%) | 1 (25.0%) | 2 (50.0%) |
| AG (*n* = 16) | 12 (75.0%) | 6 (37.5%) | 4 (25.0%) |
| AA (*n* = 16) | 11 (68.8%) | 4 (25.0%) | 5 (31.3%) |
| | | rs894160 (PLIN1) | |
| AA + AG (*n* = 17) | 15 (88.2%) | 7 (41.2%) | 5 (29.4%) |
| GG (*n* = 19) | 10 (52.6%) | 3 (15.8%) | 6 (31.6%) |
